# Advancing Thrombosis Research: A Novel Device for Measuring Clot Permeability

**DOI:** 10.3390/s24123764

**Published:** 2024-06-09

**Authors:** Elia Landi, Marco Mugnaini, Tunahan Vatansever, Ada Fort, Valerio Vignoli, Elvira Giurranna, Flavia Rita Argento, Eleonora Fini, Giacomo Emmi, Claudia Fiorillo, Matteo Becatti

**Affiliations:** 1 Department of Information Engineering and Mathematics, University of Siena, 53100 Siena, Italy; marco.mugnaini@unisi.it (M.M.); t.vatansever@student.unisi.it (T.V.); ada.fort@unisi.it (A.F.); valerio.vignoli@unisi.it (V.V.); 2 Department of Experimental and Clinical Biomedical Sciences “Mario Serio”, University of Firenze, 50121 Florence, Italy; elvira.giurranna@unifi.it (E.G.); flaviarita.argento@unifi.it (F.R.A.); eleonora.fini@unifi.it (E.F.); claudia.fiorillo@unifi.it (C.F.); matteo.becatti@unifi.it (M.B.); 3 Department of Medical, Surgical and Health Sciences, University of Trieste, 34100 Trieste, Italy; giacomo.emmi@units.it

**Keywords:** blood clot, permeability measurement, low-cost, portable measurement system

## Abstract

Thromboembolism, a global leading cause of mortality, needs accurate risk assessment for effective prophylaxis and treatment. Current stratification methods fall short in predicting thrombotic events, emphasizing the need for a deeper understanding of clot properties. Fibrin clot permeability, a crucial parameter in hypercoagulable states, impacts clot structure and resistance to lysis. Current clot permeability measurement limitations propel the need for standardized methods. Prior findings underscore the importance of clot permeability in various thrombotic conditions but call for improvements and more precise, repeatable, and standardized methods. Addressing these challenges, our study presents an upgraded, portable, and cost-effective system for measuring blood clot permeability, which utilizes a pressure-based approach that adheres to Darcy’s law. By enhancing precision and sensitivity in discerning clot characteristics, this innovation provides a valuable tool for assessing thrombotic risk and associated pathological conditions. In this paper, the authors present a device that is able to automatically perform the permeability measurements on plasma or fibrinogen in vitro-induced clots on specific holders (filters). The proposed device has been tailored to distinguish clot permeability, with high precision and sensitivity, between healthy subjects and high cardiovascular-risk patients. The precise measure of clot permeability represents an excellent indicator of thrombotic risk, thus allowing the clinician, also on the basis of other anamnestic and laboratory data, to attribute a risk score to the subject. The proposed instrument was characterized by performing permeability measurements in plasma and purified fibrinogen clots derived from 17 Behcet patients and 15 sex- and age-matched controls. As expected, our results clearly indicate a significant difference in plasma clot permeability in Behcet patients with respect to controls (0.0533 ± 0.0199 d vs. 0.0976 ± 0.0160 d, *p* < 0.001). This difference was confirmed in the patient’s vs. control fibrin clots (0.0487 ± 0.0170 d vs. 0.1167 ± 0.0487 d, *p* < 0.001). In conclusion, our study demonstrates the feasibility, efficacy, portability, and cost-effectiveness of a novel device for measuring clot permeability, allowing healthcare providers to better stratify thrombotic risk and tailor interventions, thereby improving patient outcomes and reducing healthcare costs, which could significantly improve the management of thromboembolic diseases.

## 1. Introduction

Thromboembolism, the leading cause of mortality worldwide, is characterized by the formation of obstructive intravascular clots (thrombi) and their mechanical breakage (embolization) [1]. Current risk stratification strategies for thromboembolism have low predictive value in asymptomatic subjects classified as intermediate cardiovascular risk, and the inaccurate stratification provided by existing models may potentially result in individuals being either exposed to excessive or insufficient prophylaxis [2].

Fibrin is a major component of both venous and arterial thrombi. Our previous studies revealed that individuals with hypercoagulable states exhibited compact fibrin clots resistant to lysis [3,4,5,6,7]. Fibrin clot permeability, which reflects an average pore size between fibrin fibers, currently represents the most used parameter, measured by standardized hydrostatic pressure-driven assay, to estimate fibrin clot structure in different disease conditions [8]. Reduced fibrin clot permeability has been reported to be associated with the recurrence of thrombotic events [9,10,11,12,13,14,15].

Healthcare providers typically conduct a risk assessment to determine whether a patient is at high, moderate, or low risk, which typically involves administering questionnaires that collect information about the patient’s age, medical history, medications, and relevant lifestyle factors [16,17,18,19,20,21]. Nevertheless, this method seems inadequate. A pilot international study focusing on standardizing fibrin clot measurement highlighted the potential advantages of adopting a standardized protocol for risk assessment, indicating that clot permeability measurement could be useful in clinical settings, particularly if implemented in automated analyzers [22].

Here, we propose a portable and cost-effective permeability measurement system based on the findings reported in [23]. This system is designed to identify individual thrombotic risk and has the potential to contribute greatly to improved patient outcomes in endothelial dysfunction cardiovascular diseases, blood-brain barrier permeability, and glaucoma drainage devices [24,25,26]. 

The proposed device is designed to measure the permeability of clots formed in vitro, using cost-effective disposable filters to support the clots. The clot and its support are aligned with a reservoir where a liquid is maintained at constant pressure using a pneumatic system. The flow of liquid through the clot over time is then quantified using a cantilever balance, therefore enabling the estimation of thrombus permeability. The proposed clot permeability measurement assay represents an excellent indicator of the individual propensity to thrombosis for clinicians, who, in conjunction with other anamnestic and laboratory data, can assign to patients a risk score. The device and the disposable filters have been assayed in terms of performance concerning mass measurement and filter permeability, excluding clot presence. Subsequently, the device’s functionality has been validated using biological samples, specifically fibrin clots purified from plasma derived from both healthy individuals and patients with high cardiovascular risk.

## 2. Materials and Methods

**Figure 1:**
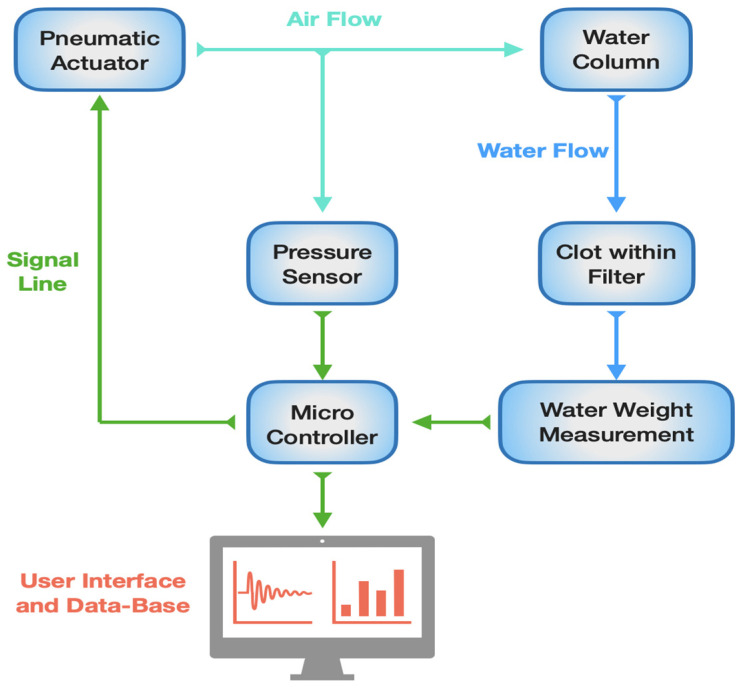
Block diagram of clot permeability measurement system. Green lines represent the signal line and the communication between the different subsystems. Light-blue lines represent the airflow from the pneumatic actuator (a pump in this case). Blue lines represent the water flow from the water column.

The proposed measurement system, as illustrated in Figure 1, is designed to assess clot permeability. In fluid mechanics, permeability refers to the ability of a porous material to allow fluid to flow through its structure. The operational concept of the proposed device involves positioning a column of consistently pressurized water above the sample whose permeability is to be determined. Sample permeability is then evaluated by monitoring the mass of the liquid that percolates through it over time, given that the density of the liquid is known. In the case of interest, the sample holder consists of a disposable filter with a LUER connection, where the clot is placed. The mechanical support provided by the filter is essential to prevent the clot from detaching from it. 

Regarding the diagnostic application of the device, efforts were made to minimize the amount of biological samples required for permeability measurement. Specifically, the disposable support filter can hold a maximum of 35 µL of biological sample, which comprises the clot and other substances in the liquid phase, which are expelled from the support filter during the permeability measurement.

Since the proposed system relies on measuring the liquid mass to determine the volume of fluid that percolates through the sample, for accurate measurements, it is essential to use a liquid with a known density. The liquid phase present in the sample is difficult to characterize in terms of density and volume. Therefore, to minimize its impact on determining the fluid phase density, the instrument is designed to use ultrapure water in a volume significantly greater—by over an order of magnitude—than that of the biological sample. This approach reduces the uncertainty of the percolated liquid’s density, assuming it to be that of pure water.

### 2.1. Mathematical Model

Permeability can be measured by exploiting Darcy’s law reported in Equation (1), which relates the volume flow of a liquid flowing through a porous medium sample to the sample geometry, to the pressure applied at the sample interface, and to the liquid characteristics.

(1) $$Q\left(t\right)=\frac{K_{s}A\Delta p(t)}{\mu L}$$

The quantities in Equation (1) are defined in Table 1.

From (1), it is possible to obtain the permeability $K_{s}$ by measuring the volume flow Q(t) and pressure Δp over time. Since the measurement of the volume flow is relatively complicated, especially considering the small quantities involved in the measurement of interest, it is convenient to consider the percolated liquid volume, *V*(*t*), over time:

(2) $$\frac{dV\left(t\right)}{dt}=\frac{K_{s}A\Delta p(t)}{\mu L}$$

By considering a steady state situation where the pressure gradient and the flow are constant, it is possible to obtain the permeability value as:

(3) $$K_{s}=\frac{\mu L}{A\Delta p}\frac{V\left(t\right)}{t-t_{0}}$$

where $t_{0}$ represents the initial time. Knowing the density of the liquid used to carry out the measurement, it is possible to obtain the permeability of the sample by measuring the mass of the percolated liquid:

(4) $$K_{s}=\frac{\mu L}{A\Delta p\rho }\frac{dM}{dt}=\frac{\mu L}{A\Delta p\rho }\frac{M\left(t\right)}{t-t_{0}}$$

where M(t) is the mass of the percolated liquid and *ρ* is the liquid density. In this way, it is possible to obtain the permeability of the sample by measuring the mass of the percolated liquid over time instead of the flow, reducing the complexity of the measurement system.

Notice that, even by keeping the pressure constant, the liquid discharge does not occur at a constant rate; rather, in drops, i.e., it is intermittent because of surface tension effects or other fluid properties. Therefore, Equation (1) represents the average flow through the clot. To accurately measure the non-continuous flow in real conditions, the observation time needs to be long enough to capture a significant number of drops, ensuring an accurate representation of the overall (average) discharge behavior.

The comprehensive assessment of the measurement system uncertainty can be evaluated under the assumption of non-correlation among individual sources of uncertainty, referring to Equation (4) by the following relationship:

(5) $$u\left(K_{s}\right)=\sqrt{\sum_{i}{\left(\frac{\partial K_{s}}{\partial x_{i}}\right)}^{2}u^{2}\left(x_{i}\right)}$$

where each $x_{i}$ indicates each individual measurand in (4) and therefore we obtain:

(6) $$u\left(K_{s}\right)=\sqrt{\begin{array}{c} {\left(\frac{\mu L}{A\Delta pt\rho }\right)}^{2}u^{2}\left(M\right)+{\left(\frac{ML}{A\Delta pt\rho }\right)}^{2}u^{2}\left(\mu \right)+{\left(\frac{M\mu }{A\Delta pt\rho }\right)}^{2}u^{2}\left(L\right)+{\left(-\frac{M\mu L}{A^{2}\Delta pt\rho }\right)}^{2}u^{2}\left(A\right)+\\ {\left(-\frac{M\mu L}{A\Delta p^{2}t\rho }\right)}^{2}u^{2}\left(\Delta p\right)+{\left(-\frac{M\mu L}{A\Delta pt^{2}\rho }\right)}^{2}u^{2}\left(t\right)+{\left(-\frac{M\mu L}{A\Delta pt\rho^{2}}\right)}^{2}u^{2}\left(\rho \right) \end{array}}$$

The permeability uncertainty obtained by exploiting Equation (6) is reported in Section 3 for each measurement.

The calculation of the uncertainty of the permeability measurements is necessary to evaluate the overall measurement quality. Since there is no gold standard for this type of measurement, it seems mandatory to quantify both the uncertainty of the instrument measurements and the variability of clot preparation. 

### 2.2. Study Population 

Seventeen patients with Behçet syndrome who attended the Florence Behçet Center and 15 age-matched healthy control subjects were included in the study. All the patients were diagnosed as having Behçet disease according to International Study Group criteria [27]. Patients with other autoimmune diseases, active infections, or neoplastic conditions were excluded. Blood samples were collected from patients without immunosuppressive therapy, and only prednisone assumption < 10 mg/d was allowed. No colchicine-treated patients were enrolled. The study protocol was approved by the local Ethical Committee, and informed consent was obtained from all subjects enrolled. The study was approved on 31/05/2022 by Comitato Etico Regionale per la Sperimentazione Clinica della Regione Toscana, Project: “GUt Dysbiosis and cardioVascular Involvement in BEhçet Syndrome” (GUDVIBES), number: 21395_bio. The sample size was based on paired tests for continuous outcomes, assuming an increase in fibrin degradation rate of 80% with respect to controls, with 80% power, based on the fibrin degradation values presented previously [3].

### 2.3. Blood Sample Collection

Blood samples were collected from patients without immunosuppressive therapy, and only prednisone assumption < 10 mg/d was allowed.

Blood samples were collected from patients who had experienced a myocardial infarction at least six months prior to sample collection. Vacutainer tubes containing 0.109 mol/L buffered trisodium citrate (1:10). After centrifugation (1500× *g* for 15 min at 4 °C), aliquots of plasma were used for experiments and for fibrinogen purification.

### 2.4. Fibrinogen Purification 

Fibrinogen was purified from patients and controls using the previously described ethanol precipitation method [3]. Fibrinogen concentration was determined spectrophotometrically at 280 nm (the extinction coefficient 1.51 mg/mL was used). 

### 2.5. Clot Preparation 

Clots were generated either by incubating 10 μL of human thrombin (10 U/mL-T6884 SIGMA) with 20 μL of plasma for 1 h at 25 °C or by incubating 15 μL of human thrombin (5 U/mL-T6884 SIGMA) with 15 μL of purified fibrinogen (0.4 mg/mL) in 100 mM Tris/HCl, 5 mM CaCl_2_, pH 7.4, for 1 h at 25 °C. 

In both plasma or fibrinogen clots, 30 μL samples were transferred using pipettes into hydrophobic membrane syringe filters used as supports for performing the measurements. In particular, syringe filters in hydrophobic Polytetrafluoroethylene (PTFE). With 0.22 μm and 0.45 μm meshes, a diameter of 13 mm, and LUER connection were chosen.

### 2.6. Measurement Protocol and Measurement System

To assess permeability, a predetermined amount of pure distilled water was placed above the filter containing the biological sample. The measurement liquid volume was set at 400 μL to ensure that the potential presence of any residual fluid in the biological sample was not significant. This approach was aimed at minimizing the effects due to potential density discrepancies between the two fluids.

**Figure 2:**
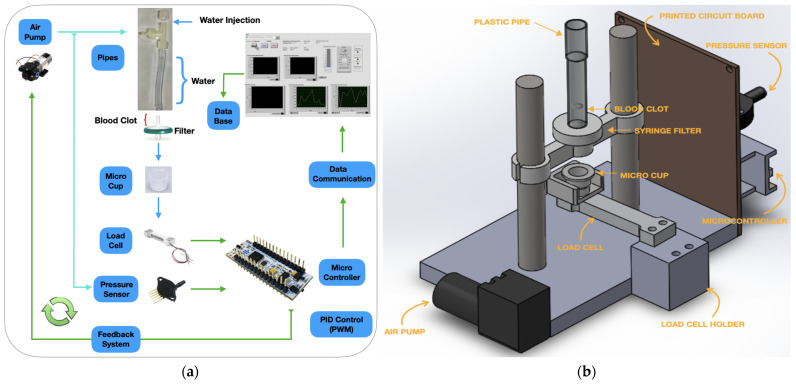
(**a**) Block diagram of the clot permeability measurement system; (**b**) 3D representation of the instrument mechanical realization.

The measurement system is based on a novel in-house designed standalone instrument comprising a pneumatic circuit, which includes a pump to set the pressure of the liquid above the biological sample, a pressure sensor to measure the pressure inside the pneumatic circuit and a precision scale based on a load cell to measure mass of the liquid percolated through the biological sample. An STM32L432KC microcontroller (STMicroelectronics) was exploited to manage the instrument, control the pressure with a feedback loop, and acquire the signals from the sensors. In Figure 2a. the detailed block diagram of the developed measurement setup with the electronic, pneumatic, and hydraulic circuits is shown. The representation of the user interface is also shown. A tailored mechanical structure supporting the sample, hosting the pneumatic circuit, the scale, and the electronics was developed (Figure 2).

The design of the pneumatic system, in terms of maximum pressure, was carried out considering the dynamics of blood pressure in the human body assumed to be between 60 mmHg–90 mmHg (11.9/7.9 kPa) and 120/80 mmHg (15.9/10.6 kPa).

Following this consideration, the system was sized to provide a maximum pressure of 10 kPa on the surface of the liquid column.

A tube with a section of 3 mm and a length of 10 cm, capable of containing approximately 400 μL of liquid, was used. Hence, the length of the liquid column makes the influence of hydrostatic pressure at the interface with the biological sample negligible.

A NMP05-KPDC-S (KNF) diaphragm pump, capable of providing a maximum pressure of 40 kPa, driven by a DC motor, was used. The pressure measurement in the pneumatic circuit was carried out by means of a differential piezoresistive sensor, MPX5010DP (Freescale NXP), capable of supporting pressures up to 40 kPa, which guarantees a full scale of 10 kPa and a maximum error of 0.5 kPa. The pump is controlled in feedback exploiting the pressure measurement inside the pneumatic circuit; in particular, an ON/OFF controller was implemented.

Due to the small quantity of liquid percolated from the sample, mass measurement should be performed at high resolution, ideally around 10 mg. A cantilever load cell was used, which couples ease of placement and high resolution. In particular, an SMD3277-010 (Strain Measurement Devices, Chedburgh Bury St Edmunds, England) bridge load-cell-based mass sensor having a full scale of 10 g was adopted. The larger full scale compared to the quantity of liquid to be measured is mandatory because support is positioned on the force sensor, which allows the attachment of a disposable micro cup collecting the liquid percolated from the sample. The adopted mass sensor has a rated output of 1 mV/V and a nonlinearity error of less than 0.05% with respect to the rated maximum load (i.e., <0.005 g) in the range for the application.

The load cell bridge is powered with a 5 V voltage reference, and the output signal of the bridge is subsequently amplified with an instrumentation amplifier with a gain of 500 *V*/*V* in order to correctly exploit the dynamics of the analog-digital converter of the microcontroller. As far as drift is concerned, the accurate front-end electronic design, based on precision differential amplifiers, offset compensation circuits, and regulated reference voltages, ensures a small offset; therefore, the drift spans some μV, which corresponds to a few mg.

The STM32 L432KC microcontroller is also used for signal acquisition from the sensors and for serial communication with a PC. Through serial communication, it is possible to set the pressure inside the pneumatic circuit and read the pressure and mass parameters in real-time. A LabVIEW interface on the PC allows for data acquisition, saving, and instrument settings. Moreover, the VI, at the beginning of each measurement, allows for offset and tare compensation (removed by an offset null procedure).

### 2.7. System Characterization

To characterize the developed instrument, the first tests concerned ensuring a stable measurement of the mass, observing the output of the mass sensor for periods lasting about the duration of a complete measurement. The performance expected from the design was confirmed; in fact, drifts in the order of a few milligrams are observed over periods of some tens of minutes. 

**Figure 3:**
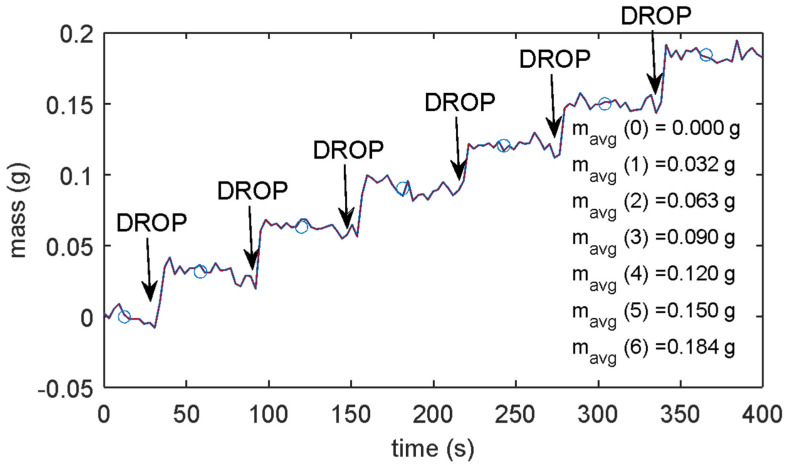
Drop test results: measured mass with 30 µL of distilled water subsequently dispensed into the cup. The markers represent the average masses measured for the individual drops, the numerical values of which are reported in the figure.

Then, the resolution of the mass measurement system was assessed: multiple tests were conducted by introducing known volumes of liquid directly into the disposable microcup. The mass measurement precision resulted in better than 0.01 g and was evaluated as an extended A type uncertainty, with a coverage factor 2, i.e., two times the standard deviation of the obtained measurements in time periods ten minutes long, i.e., repeating the measurement in identical conditions (approximately on constant masses, at the same temperature and environmental conditions) about one hundred times. The instrument resolution was evaluated as three times the estimated precision, i.e., 0.03 g. To confirm the assessed mass resolution, tests were conducted by introducing small volumes of liquid with mass 0.03 g directly in the disposable micro cup at fixed time intervals. For instance, Figure 3 shows the results obtained with an experiment in which droplets of 30 µL of distilled water were subsequently dispensed into the cup.

A laboratory class A pipette (VWR International Srl, Milan, Italy) with a full scale of 1 mL was used for the purpose.

Figure 3 shows the mass measurement in a test where six droplets were consequently added to the disposable micro-cup; the mass increments are distinguishable from the noise floor, confirming the estimated resolution.

The whole measurement technique uncertainty must be assessed to ensure measurement significance. To this aim, apart from the uncertainty of the electronic instrument, which is assessed by exploiting Equation (6), the uncertainty related to the reproducibility of the clot supports (different permeability of individual filters) and of the clot preparation were evaluated [23]. In the measurement system, in fact, the permeability that is measured refers to the two media that the test liquid passes through, i.e., the clot and the filter. In the preliminary characterization of the measurement setup, the influence of the used clot supports on the permeability measurements, as well as variations in permeability between different supports with the same construction characteristics, was studied. Different filters with both meshes were tested to verify their permeability and to analyze the variations in permeability between filters having the same characteristics in terms of mesh. 

**Figure 4:**
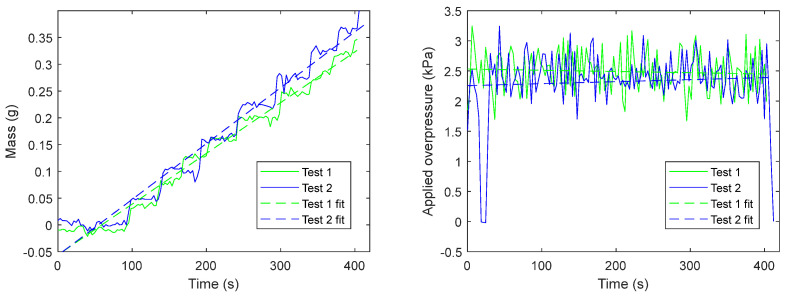
Experimental results were obtained with a pristine 0.22 µm filter.

The support filters were tested using the measurement protocol above described with different applied pressures: about 2.5 kPa for the filters with a 0.45 µm mesh and 3 kPa for those with a 0.22 µm mesh; 10 different samples of each filter type were tested. To obtain the permeability measurement, a linear fitting of mass data over time was used since, as previously described, the liquid flow is not constant, as shown in Figure 4, depicting two typical liquid discharge trends over time using 0.22 µm mesh filters. The linear regression considers data from the instant in which the first drop fell to the instant in which the last drop fell.

The slope of the linear fitting represents the average $\frac{dM\left(t\right)}{dt},$ hence, it can be used in Equation (4) to assess the sample permeability. 

The average permeabilities of the 0.22 µm and of the 0.45 µm mesh filters were 0.32 d ± SD and 0.46 d ± SD, respectively (a filter thickness of 1 mm was considered). The permeability values are expressed in Darcy (1 d = 9.869,·10^−13^ m^2^). Therefore, for the same filter type, it is possible to carry out clot permeability measurements without performing a priori measurement of bare filter permeability. As expected, the denser mesh filter presents lower permeability values, and the ratio between the two permeability values is consistent with the two mesh pore sizes.

**Figure 5:**
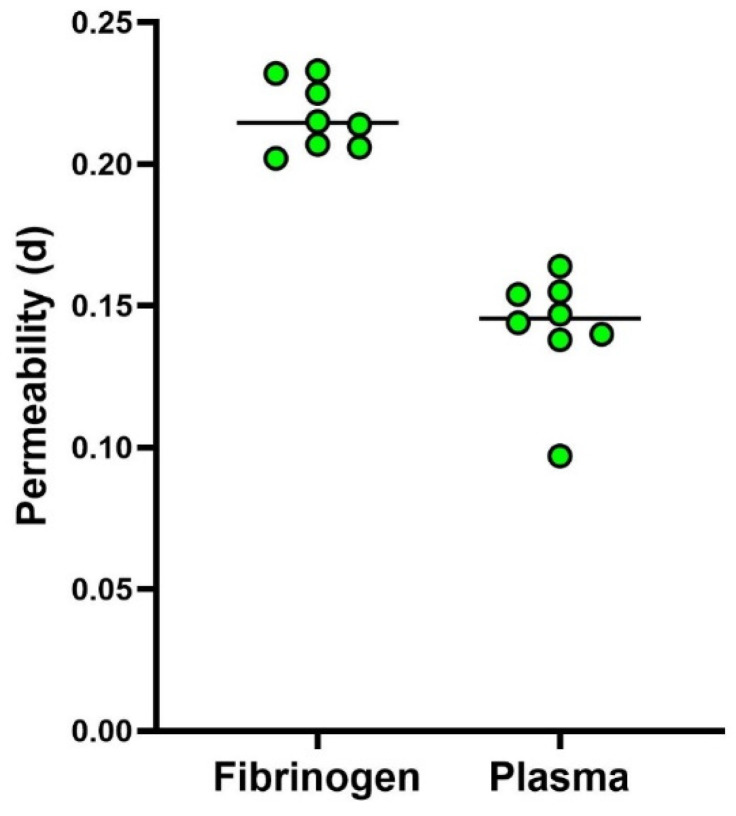
Data were obtained on fibrinogen and plasma clots prepared from a single blood sample taken from one subject.

Finally, to characterize clot reproducibility in a single healthy subject, eight fibrinogen clots and eight plasma clots were prepared and assayed with 0.22 µm filters. For plasma clots, the median permeability value resulted in 0.1455 (range 0.0970–0.0670) d, and for the fibrinogen clots resulted in 0.2145 (range 0.2020–0.2330) d. The results of these experiments are shown in Figure 5, where the black line represents the median value.

## 3. Results

The system was tested with fibrin clots obtained from purified fibrinogen and from plasma, from healthy subjects, and from high cardiovascular-risk patients. Both types of filters were used; in particular, the 0.45 µm mesh filters for the plasma-derived clots and the 0.22 µm mesh filters for the fibrinogen-derived clots were used on the basis of previously obtained data [25].

All measurements were performed following the measurement protocol described in Section 2 and were stopped when at least 85% of the water was discharged.

As reported in Section 2.4, clot permeability measurements were obtained by exploiting the linear fitting of the mass over time, starting from the first measured drop.

Continuous variables were described as mean values and standard deviation (SD). Data distribution was checked using the Shapiro-Wilk test. An Independent *t*-test for unpaired data was used for data comparison between patients and controls. A *p*-value of <0.05 was considered statistically significant. Statistical analyses were performed using the GraphPad Prism software version 6.01 (GraphPad Software, San Diego, CA, USA).

### 3.1. Plasma Clots Measurement Results

Plasma clots from 15 healthy subjects and from 17 Behcet patients were tested.

As shown in Table 2, the measurement results obtained with the control clots gave an average permeability of 0.10 d, with an average deviation with respect to the average permeability over the different clots of 13.41%., while the SD is about 0.02 d. In the proposed setup, the uncertainty of the measurements related to the instrument (as per Equation (6)) is, on average, an order of magnitude lower with respect to the measurement dispersion; this means that the variability comes from sample preparation and sample variability and that the instrument provides accurate enough measurements for the application of interest.

As reported in Table 3, average patient clot permeability measurements resulted in 0.053 d, which is significantly lower compared to permeability values of control clots (0.100 d). As expected, in patients, a higher relative dispersion of measurements is observed (mean deviation magnitude of about 70% and SD of about 0.02 d). These differences can be attributed to the inherent differences in patient health conditions.

**Figure 6:**
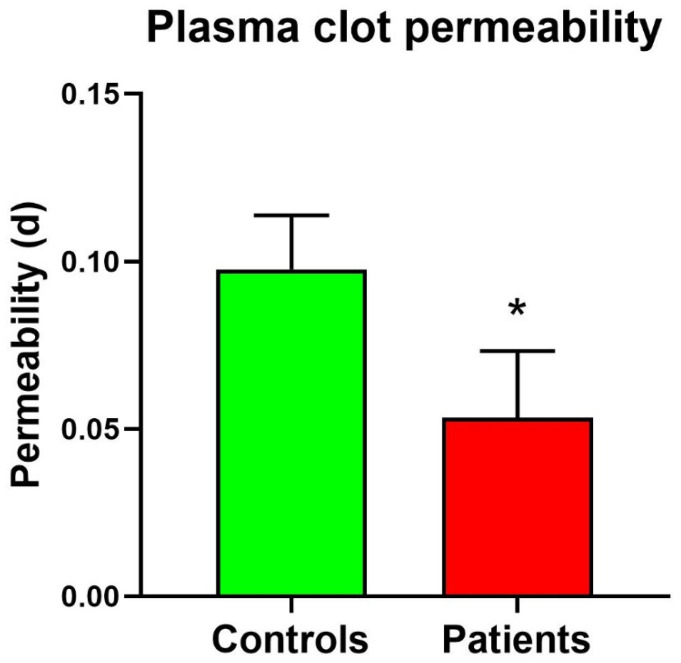
Plasma Clot permeability in patients and controls. * Significant difference vs. controls at the *p* < 0.05 level.

*t*-test analysis between “Plasma Control clots” and “Plasma Patient clots” permeability was performed. The results indicate a significantly lower permeability exhibited by patient plasma clots compared to plasma control clots. Indeed, the calculated average permeability value for patient plasma clots results in 0.100 d, while the corresponding value for patient plasma clots results in 0.053 d, suggesting that the proposed clot permeability measurement system is able to distinguish controls from patients (Figure 6).

The system demonstrated consistent and reliable performance in detecting permeability in healthy subjects and patients, showing a significant difference in permeability, with patient clots exhibiting lower permeability values. This observation aligns with expectations and underscores the system’s capability to detect subtle differences in clot permeability among various samples.

### 3.2. Fibrin Clot Measurement Results

The above-described measurement protocol was replicated using clots obtained from purified fibrinogen from healthy subjects and Behcet patients. As already mentioned, in this case, 0.22 µm pore size filters were used. More in detail, nine controls and seven patients were analyzed.

Table 4 shows the measurement obtained on control fibrin clots. The average permeability is 0.117 d, and as previously described, measurements have been taken considering different overpressures.

The same procedure was carried out for the patient's fibrin clot. Experiment results are presented in Table 5.

As indicated, the permeability values of patient fibrin clots ranged from 0.027 d to 0.071 d, with a calculated average permeability value of 0.049 d.

**Figure 7:**
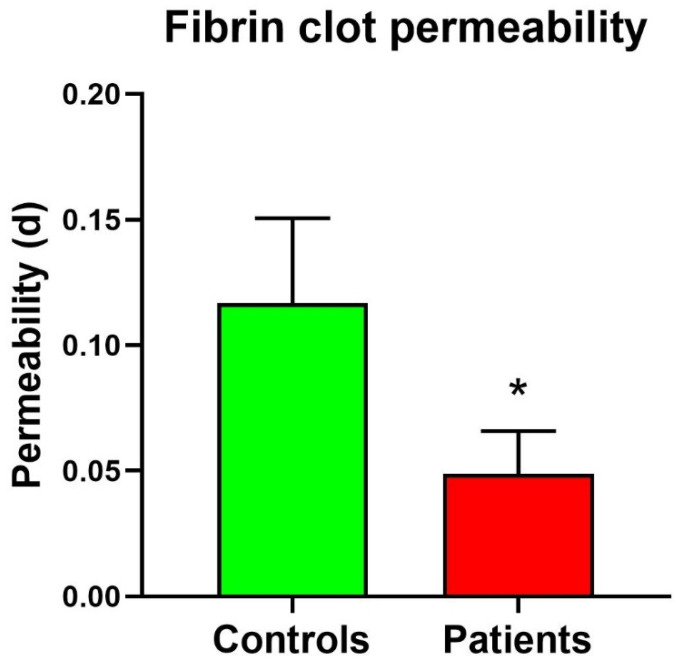
Fibrinogen clot permeability in patients and controls. * Significant difference vs. controls at the *p* < 0.05 level.

A *t*-test comparison between control fibrin clots and patient fibrin clots in terms of permeability was conducted. The calculated average permeability value for patient fibrin clots was 0.049 d, whereas for control fibrin clots was 0.117 d. Thus, the results indicate again that it is possible to discriminate between the permeability of clots from controls and from patients. The box plot of control fibrin clot and patient fibrin clot is represented in Figure 7.

## 4. Discussion

Thromboembolism, the leading cause of mortality worldwide, is characterized by the formation of obstructive intravascular clots (thrombi) and their mechanical breakage (embolization), leading to severe cardiovascular conditions such as myocardial infarction and stroke [28]. Current risk stratification methods fail to accurately predict thrombotic events, leading to inappropriate prophylaxis exposing individuals to either excessive bleeding risks or thromboembolic events [2]. Emerging evidence suggests that the formation of more compact clots, which are relatively resistant to lysis, may predispose individuals to arterial and venous thromboembolism [10,11,29]. A prothrombotic clot, generally characterized by a denser fibrin clot with respect to healthy individuals, shows a smaller pore size and thicker fibers in fibrin networks, a low permeability coefficient, and a reduced plasma clot lysis time [10]. In general, fibrin clots with thinner fibers and smaller pores are more compact and less permeable, whereas clots with thicker fibers are more permeable and more susceptible to fibrinolysis [30]. These changes in fibrin clot features have been observed not only in patients with thromboembolism but also in various diseases associated with an increased risk of thromboembolic events, including cancer, diabetes, antiphospholipid syndrome, and inflammatory diseases [11,30,31].

Fibrin clot porosity is typically estimated by measuring clot permeability under different hydrostatic pressures using different reagents and concentrations. Clot permeability is calculated based on the volume of a buffer flowing through a fibrin gel over a specific time using the Darcy constant (Ks). Today, various methods and models are used to determine fibrin clot porosity, including those where clotting is initiated by thrombin or tissue factor in platelet-poor or platelet-rich plasma, utilizing manual or semi-automated techniques [8,32,33]. Given the importance of measuring fibrin clot permeability, the Factor XIII and Fibrinogen Subcommittee of the Scientific and Standardization Committee of the International Society on Thrombosis and Haemostasis published the first recommended protocol for Ks measurement in 2012 [22]. Indeed, universal standards are essential to ensure acceptable variability between laboratories.

Our study introduces a novel system for measuring blood clot permeability, utilizing a pressure-based approach adhering to Darcy’s law. This system aims to enhance the precision and sensitivity in discerning clot characteristics, which is critical for improving thrombotic risk assessment. Our device is designed to automatically measure the permeability of clots formed in vitro on specific holders and was validated by conducting measurements on both plasma and fibrinogen clots from patients and healthy individuals. As expected, our preliminary findings revealed that clots from Behcet patients exhibited significantly lower permeability compared to those from controls, consistent with previous reports linking hypercoagulable states to more compact and resistant fibrin clots [31]. The ability to accurately measure clot permeability and thereby assess individual thrombotic risk is crucial for developing personalized prophylactic and therapeutic strategies. This is particularly important given the limitations of existing risk stratification models, which often fail to account for the complex interplay of genetic, environmental, and clinical factors influencing thrombus formation and stability [16]. Moreover, the development of more reliable and accessible diagnostic tools can potentially bridge the gap in clinical practice, where there is a need for more precise risk stratification to guide treatment decisions. The precision and repeatability of measurements of our proposed device are enhanced by the system’s design, which minimizes the influence of external variables. By providing more accurate and repeatable measurements of clot permeability, the proposed system can aid healthcare providers in better stratifying thrombotic risk and tailoring interventions, thereby improving patient outcomes and reducing healthcare costs. Furthermore, the significant difference in permeability between Behcet patients and control clots highlights the device’s sensitivity in detecting pathological changes in clot structure. This capability is particularly relevant for managing conditions associated with increased thrombotic risk [7,11,12].

Our device is a portable, cost-effective solution designed for clinical settings, emphasizing accessibility and ease of use, with automated, software-based calculations to measure blood clot permeability using a pressure-based approach. Permeability measurement is conducted by applying overpressure on the surface of the clot and measuring the mass of percolated water over time. The measurement duration ranges from approximately 60 s to a maximum of 1800 s in low permeability clots. When comparing these results with other systems based on the same measurement technique, such as the method described by Ząbczyk et al. [33], there is an improvement of at least one order of magnitude in terms of measurement time. This significant reduction in measurement time is achieved by the use of a clot support that provides structural integrity to the clot, allowing for the application of greater pressure during testing. The increased pressure capability accelerates the measurement process. Furthermore, the proposed device utilizes off-the-shelf components, which help to reduce the overall cost of the instrument compared to the system described by Ząbczyk et al. [33]. This cost-efficiency, combined with the enhanced performance, makes the new device a practical and economical alternative for fibrin clot permeability assessment.

Our study has several limitations. Firstly, it involved a relatively small sample size, with 17 patients and 15 controls. This limited number of participants may not fully capture the variability in clot permeability across different populations. Future studies should include a larger and more diverse cohort to validate the findings and enhance the generalizability of the results. Moreover, the process of clot preparation can introduce variability, especially when different operators are involved. Although efforts were made to standardize the procedure, slight differences in the preparation and handling of samples could affect the results. Further refinement and automation of the clot preparation process could help minimize this variability. It should be noted, however, that the clot permeability measurements were performed on clots formed in vitro. While this method allows for controlled conditions, it may not entirely replicate the complex environment within the human body where multiple physiological factors influence clot formation and stability. Future research should aim to correlate in vitro findings with in vivo data to ensure the clinical relevance of the measurements.

## 5. Conclusions

Our study demonstrates the feasibility and efficacy of a novel device for measuring clot permeability, which could significantly improve the management of thromboembolic diseases.

Given the limitations of existing risk stratification models, which often fail to account for the complex interplay of genetic, environmental, and clinical factors influencing thrombus formation and stability, the ability to accurately measure clot permeability and thereby assess individual thrombotic risk is crucial for developing personalized prophylactic and therapeutic strategies. The precision and repeatability of our proposed device’s measurements are enhanced by its design, which minimizes the influence of external variables.

The proposed device, which automates permeability measurements on plasma or fibrinogen clots in vitro, was thoroughly characterized from a metrological perspective. It was validated through a measurement campaign involving clots derived from both patients and healthy controls. The results showed that clots from high cardiovascular-risk patients had significantly lower permeability than those from healthy individuals, aligning with previous research that associates hypercoagulable states with more compact, less permeable fibrin clots.

While this study involved a relatively small sample size, the results are promising and indicate the device’s potential to significantly impact clinical practice. Future research should focus on further validating this device in larger, more diverse cohorts and exploring its integration into routine clinical practice to enhance patient outcomes.

## Figures and Tables

**Table 1:** Parameters of permeability equation.

Symbol	Quantity	Measurement Unit
*A*	Sample Cross-Sectional Area	($m^{2}$)
*L*	Clot Length	(m)
*μ*	Liquid Dynamic Viscosity	(Pa × s)
*Q*(*t*)	Discharge rate	($m^{3}/s$)
Δ*p*	Pressure Difference (pressure above the sample-pressure below the sample)	(kPa)
$K_{s}$	Permeability	($m^{2}$)
*V*	Sample Volume	(m^3^)

**Table 2:** Plasma clot permeability data from healthy subjects (controls).

Clot	Dropped Water (Total)	Overpressure (Avg)	Elapsed Time	Permeability	Relative Deviation (with Respect to Avg Permeability) %	Permeability Value Uncertainty
1	0.35 g	2.8 kPa	471 s	0.109 d	10.5%	0.0083 d
2	0.36 g	3.3 kPa	525 s	0.083 d	−18.1%	0.0052 d
3	0.28 g	3.3 kPa	293 s	0.101 d	3.6%	0.0071 d
4	0.33 g	4.5 kPa	324 s	0.079 d	−23.7%	0.0056 d
5	0.25 g	3.3 kPa	377 s	0.076 d	−29.0%	0.0057 d
6	0.41 g	3.5 kPa	545 s	0.086 d	−14.0%	0.0051 d
7	0.40 g	3.3 kPa	435 s	0.116 d	15.6%	0.010 d
8	0.40 g	4.0 kPa	344 s	0.091 d	−6.9%	0.0092 d
9	0.38 g	3.4 kPa	611 s	0.081 d	−20.0%	0.0056 d
10	0.38 g	3.4 kPa	433 s	0.131 d	25.5%	0.011 d
11	0.40 g	4.8 kPa	359 s	0.095 d	−2.3%	0.0051 d
12	0.32 g	3.3 kPa	394 s	0.109 d	10.2%	0.0072 d
13	0.44 g	3.4 kPa	605 s	0.095 d	−2.5%	0.0070 d
14	0.36 g	3.3 kPa	406 s	0.117 d	16.4%	0.0092 d
15	0.30 g	4.8 kPa	287 s	0.095 d	−3.2%	0.0058 d
Average	0.37 g	3.6 kPa	481 s	0.098 d	13.4%	0.0071 d

**Table 3:** Plasma clot permeability data from high cardiovascular risk patients.

Clot	Dropped Water (Total)	Overpressure (Avg)	Elapsed Time	Permeability	Relative Deviation (with Respect to Avg Permeability) %	Permeability Value Uncertainty
1	0.35 g	4.5 kPa	853 s	0.041 d	−29.0%	0.0032 d
2	0.32 g	3.2 kPa	660 s	0.072 d	27.0%	0.0053 d
3	0.33 g	4.4 kPa	1062 s	0.035 d	−51.4%	0.0027 d
4	0.33 g	4.4 kPa	558 s	0.052 d	−0.4%	0.0041 d
5	0.39 g	4.4 kPa	763 s	0.057 d	6.9%	0.0043 d
6	0.34 g	3.2 kPa	633 s	0.075 d	29.3%	0.0054 d
7	0.37 g	4.5 kPa	660 s	0.062 d	14.9%	0.0049 d
8	0.30 g	3.3 kPa	982 s	0.044 d	−19.9%	0.0034 d
9	0.41 g	3.4 kPa	615 s	0.070 d	24.8%	0.0039 d
10	0.19 g	3.4 kPa	2437 s	0.006 d	−728.1%	0.0024 d
11	0.32 g	3.3 kPa	1043 s	0.038 d	−39.5%	0.0026 d
12	0.39 g	3.3 kPa	810 s	0.069 d	24.0%	0.0051 d
13	0.35 g	3.3 kPa	760 s	0.070 d	25.0%	0.0058 d
14	0.34 g	4.9 kPa	1101 s	0.029 d	−79.7%	0.0026 d
15	0.33 g	4.3 kPa	455 s	0.069 d	24.0%	0.012 d
16	0.31 g	3.3 kPa	785 s	0.040 d	−32.5%	0.0043 d
17	0.35 g	4.8 kPa	395 s	0.077 d	31.3%	0.0051 d
Average	0.33 g	3.9 kPa	810 s	0.053 d	69.8%	0.0045 d

**Table 4:** Permeability results for control fibrin clots.

Clot	Dropped Water (Total)	Overpressure (Avg)	Elapsed Time	Permeability (Avg)	Relative Deviation (with Respect to Avg Permeability) %	Permeability Value Uncertainty
1	0.15 g	3.1 kPa	82 s	0.138 d	18.0%	0.013 d
2	0.35 g	3.1 kPa	422 s	0.096 d	18.0%	0.010 d
3	0.36 g	3.2 kPa	418 s	0.147 d	25.6%	0.012 d
4	0.33 g	3.2 kPa	553 s	0.083 d	29.1%	0.0068 d
5	0.35 g	7.0 kPa	94 s	0.082 d	29.9%	0.0065 d
6	0.36 g	3.3 kPa	366 s	0.111 d	5.1%	0.0068 d
8	0.37 g	3.7 kPa	258 s	0.183 d	56.4%	0.014 d
7	0.28 g	3.3 kPa	311 s	0.118 d	0.9%	0.0086 d
9	0.22 g	3.6 kPa	227 s	0.092 d	21.4%	0.0065 d
Average	0.33 g	4.3 kPa	230 s	0.117 d	22.7%	0.012 d

**Table 5:** Permeability results for fibrin clots from Behcet patients.

Clot	Dropped Water (Avg)	Overpressure (Avg)	Elapsed Time	Permeability (Avg)	Error (Respect to Avg Permeability) %	Permeability Value Uncertainty
1	0.38 g	3.1 kPa	225 s	0.062 d	27.5%	0.0058 d
2	0.14 g	9.9 kPa	41 s	0.046 d	6.1%	0.0043 d
3	0.31 g	3.1 kPa	1874 s	0.027 d	44.9%	0.0021 d
4	0.27 g	3.2 kPa	418 s	0.046 d	6.1%	0.0039 d
5	0.32 g	3.3 kPa	1435 s	0.028 d	42.9%	0.0021 d
6	0.34 g	3.3 kPa	422 s	0.061 d	24.5%	0.0038 d
7	0.36 g	3.7 kPa	533 s	0.071 d	44.9%	0.0047 d
Average	0.31 g	3.4 kPa	683 s	0.049 d	28.0%	0.0038 d

## Data Availability

The original contributions presented in the study are included in the article, further inquiries can be directed to the corresponding author.

## References

[1] Wendelboe A.M., Raskob G.E. (2016). Global Burden of Thrombosis: Epidemiologic Aspects. Circ. Res..

[2] Farzadfar F. (2019). Cardiovascular disease risk prediction models: Challenges and perspectives. Lancet Glob. Health.

[3] Becatti M., Emmi G., Silvestri E., Bruschi G., Ciucciarelli L., Squatrito D., Vaglio A., Taddei N., Abbate R., Emmi L. (2016). Neutrophil Activation Promotes Fibrinogen Oxidation and Thrombus Formation in Behçet Disease. Circulation.

[4] Becatti M., Marcucci R., Bruschi G., Taddei N., Bani D., Gori A.M., Giusti B., Gensini G.F., Abbate R., Fiorillo C. (2014). Oxidative modification of fibrinogen is associated with altered function and structure in the subacute phase of myocardial infarction. Arterioscler. Thromb. Vasc. Biol..

[5] Cellai A.P., Lami D., Antonucci E., Fiorillo C., Becatti M., Olimpieri B., Bani D., Grifoni E., Cenci C., Marcucci R. (2013). Fibrinolytic inhibitors and fibrin characteristics determine a hypofibrinolytic state in patients with pulmonary embolism. Thromb. Haemost..

[6] Cellai A.P., Lami D., Antonucci E., Liotta A.A., Rogolino A., Fedi S., Fiorillo C., Becatti M., Cenci C., Marcucci R. (2014). Hyperhomocysteinemia in patients with pulmonary embolism is associated with impaired plasma fibrinolytic capacity. J. Thromb. Thrombolysis.

[7] Becatti M., Emmi G., Bettiol A., Silvestri E., Di Scala G., Taddei N., Prisco D., Fiorillo C. (2019). Behçet’s syndrome as a tool to dissect the mechanisms of thrombo-inflammation: Clinical and pathogenetic aspects. Clin. Exp. Immunol..

[8] Undas A. (2016). How to Assess Fibrinogen Levels and Fibrin Clot Properties in Clinical Practice?. Semin. Thromb. Hemost..

[9] Celinska-Löwenhoff M., Zabczyk M., Iwaniec T., Plens K., Musial J., Undas A. (2018). Reduced plasma fibrin clot permeability is associated with recurrent thromboembolic events in patients with antiphospholipid syndrome. Rheumatology.

[10] Undas A. (2014). Fibrin clot properties and their modulation in thrombotic disorders. Thromb. Haemost..

[11] Undas A., Zawilska K., Ciesla-Dul M., Lehmann-Kopydłowska A., Skubiszak A., Ciepłuch K., Tracz W. (2009). Altered fibrin clot structure/function in patients with idiopathic venous thromboembolism and in their relatives. Blood.

[12] Becatti M., Mannucci A., Argento F.R., Gitto S., Vizzutti F., Marra F., Taddei N., Fiorillo C., Laffi G. (2020). Super-Resolution Microscopy Reveals an Altered Fibrin Network in Cirrhosis: The Key Role of Oxidative Stress in Fibrinogen Structural Modifications. Antioxidants.

[13] Natorska J., Ząbczyk M., Mastalerz L., Undas A. (2024). Increased factor XI but not factor XII is associated with enhanced inflammation and impaired fibrin clot properties in patients with eosinophilic granulomatosis with polyangiitis. Clin. Exp. Rheumatol..

[14] Ząbczyk M., Kruk A., Natorska J., Undas A. (2023). Low-grade endotoxemia in acute pulmonary embolism: Links with prothrombotic plasma fibrin clot phenotype. Thromb. Res..

[15] Błaż M., Natorska J., Bembenek J.P., Członkowska A., Ząbczyk M., Polak M., Undas A. (2023). Protein Carbonylation Contributes to Prothrombotic Fibrin Clot Phenotype in Acute Ischemic Stroke: Clinical Associations. Stroke.

[16] Mehta Y., Bhave A. (2023). A review of venous thromboembolism risk assessment models for different patient populations: What we know and don’t!. Medicine.

[17] Woller S.C., Stevens S.M., Jones J.P., Lloyd J.F., Evans R.S., Aston V.T., Elliott C.G. (2011). Derivation and validation of a simple model to identify venous thromboembolism risk in medical patients. Am. J. Med..

[18] Zhou H.X., Peng L.Q., Yan Y., Yi Q., Tang Y.J., Shen Y.C., Feng Y.L., Wen F.Q. (2012). Validation of the Caprini risk assessment model in Chinese hospitalized patients with venous thromboembolism. Thromb. Res..

[19] Nendaz M., Spirk D., Kucher N., Aujesky D., Hayoz D., Beer J.H., Husmann M., Frauchiger B., Korte W., Wuillemin W.A. (2014). Multicentre validation of the Geneva Risk Score for hospitalised medical patients at risk of venous thromboembolism. Explicit ASsessment of Thromboembolic RIsk and Prophylaxis for Medical PATients in SwitzErland (ESTIMATE). Thromb. Haemost..

[20] Barbar S., Noventa F., Rossetto V., Ferrari A., Brandolin B., Perlati M., De Bon E., Tormene D., Pagnan A., Prandoni P. (2010). A risk assessment model for the identification of hospitalized medical patients at risk for venous thromboembolism: The Padua Prediction Score. J. Thromb. Haemost..

[21] Patell R., Rybicki L., McCrae K.R., Khorana A.A. (2017). Predicting risk of venous thromboembolism in hospitalized cancer patients: Utility of a risk assessment tool. Am. J. Hematol..

[22] Pieters M., Undas A., Marchi R., De Maat M.P., Weisel J., Ariëns R.A. (2012). An international study on the standardization of fibrin clot permeability measurement: Methodological considerations and implications for healthy control values. J. Thromb. Haemost. JTH.

[23] Fort A., Landi E., Mugnaini M., Vatansever T., Fiorillo C., Becatti M. A portable, low cost clot permeability measurement system. Proceedings of the 2023 IEEE International Workshop on Metrology for Industry 4.0 & IoT (MetroInd4.0&IoT).

[24] Jay Widmer R., Lerman A. (2014). Endothelial dysfunction and cardiovascular disease. Glob. Cardiol. Sci. Pract..

[25] McMahon D., Bendayan R., Hynynen K. (2017). Acute effects of focused ultrasound-induced increases in blood-brain barrier permeability on rat microvascular transcriptome. Sci. Rep..

[26] Basson N., Peng C.H.S., Geoghegan P., van der Lecq T., Steven D., Williams S., Lim A.E., Ho W.H. (2024). A computational fluid dynamics investigation of endothelial cell damage from glaucoma drainage devices. Sci. Rep..

[27] Disease ISGfBs, Criteria for diagnosis of Behcet’s Disease (1990). International Study Group for Behcet’s Disease. Lancet.

[28] Satoh K., Satoh T., Yaoita N., Shimokawa H. (2019). Recent Advances in the Understanding of Thrombosis. Arter. Thromb. Vasc. Biol..

[29] Undas A., Ariëns R.A. (2011). Fibrin clot structure and function: A role in the pathophysiology of arterial and venous thromboembolic diseases. Arter. Thromb. Vasc. Biol..

[30] Siudut J., Grela M., Wypasek E., Plens K., Undas A. (2016). Reduced plasma fibrin clot permeability and susceptibility to lysis are associated with increased risk of postthrombotic syndrome. J. Thromb. Haemost..

[31] Emmi G., Becatti M., Bettiol A., Hatemi G., Prisco D., Fiorillo C. (2019). Behçet’s Syndrome as a Model of Thrombo-Inflammation: The Role of Neutrophils. Front. Immunol..

[32] Sjøland J.A. (2005). A new optimized method for the determination of fibrin clot permeability. Blood Coagul. Fibrinolysis.

[33] Ząbczyk M., Piłat A., Awsiuk M., Undas A. (2015). An automated method for fibrin clot permeability assessment. Blood Coagul. Fibrinolysis.

